# A novel etiology for automatic mode switching and ventricular noise reversion alerts: pulsed field ablation

**DOI:** 10.1016/j.hrcr.2024.08.006

**Published:** 2024-08-10

**Authors:** Joshua Lampert, Jacob Koruth, Marc A. Miller, Vivek Y. Reddy

**Affiliations:** Helmsley Center for Electrophysiology, Icahn School of Medicine at Mount Sinai, New York

**Keywords:** Pulsed field ablation, CIEDs, artifact, EMI, catheter ablation, atrial fibrillation

**Key Teaching Points**

• Pulsed Field Ablation (PFA) using a pentaspline catheter involves the application of unsynchronized therapeutic waveforms in a hierarchical set of microsecond scale biphasic pulses
• PFA can result in automatic mode switching (AMS) episodes though autogain application may alter the typical appearance of pulsed electric field applications
• PFA can also result in ventricular noise reversion alerts with a typical appearance

Artifactual recordings on cardiac implantable electronic devices (CIEDs) may be physiologic oversensing or a harbinger of lead failure with implications of requiring lead revision.^1^^,^^2^ Noise reversion is an algorithm that causes the pacing functionality of devices to switch to asynchronous pacing when there is repetitive sensing at high rates.^3^ With the recent approval from the U.S. Food and Drug Administration for two pulsed-field ablation (PFA) systems and concurrent increased uptake of the technology for catheter ablation of atrial fibrillation, artifactual recordings specific to PFA might be recorded from a CIED, warranting distinction from other environmental sources of oversensing and noise reversion alerts.

A 76-year-old male patient with a medical history of ischemic cardiomyopathy and prior substrate modification for a VT originating from left ventricular anterior/anteroseptal scar developed persistent atrial fibrillation. He had a dual-chamber implantable cardioverter defibrillator (Gallant DR, Abbott, Chicago, IL) programmed DDDR (70–100) with automatic mode switching to DDIR programmed at an atrial rate of 160 bpm. He presented for catheter ablation using the pentaspline PFA catheter (Farawave, Boston Scientific, Marlborough, MA). Device therapies were turned off before the procedure and a pre-procedure device interrogation showed normal device function and no significant events other than persistent atrial fibrillation. Upon interrogation of the device post-procedure, two automatic mode switching episodes (Figure 1) and four noise reversion alerts (Figure 2) were noted on routine post-ablation device interrogation.

**Figure 1:**
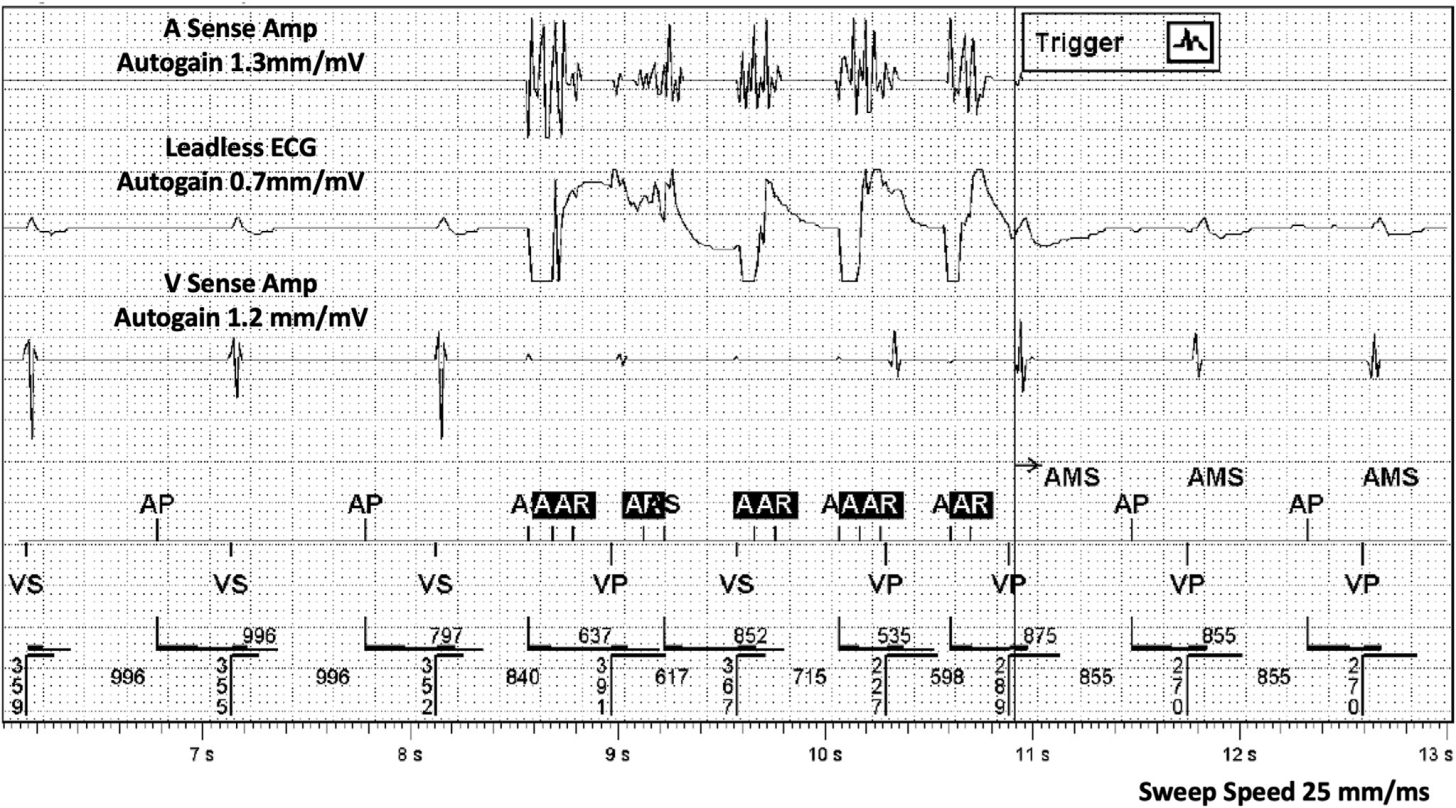
Pulsed electric field application triggering automatic mode switching. High-frequency pulses of relatively large amplitude as seen with autogain applied are noted triggering automatic mode switching to DDIR. These pulses reflect one application during pulsed field ablation.

**Figure 2:**
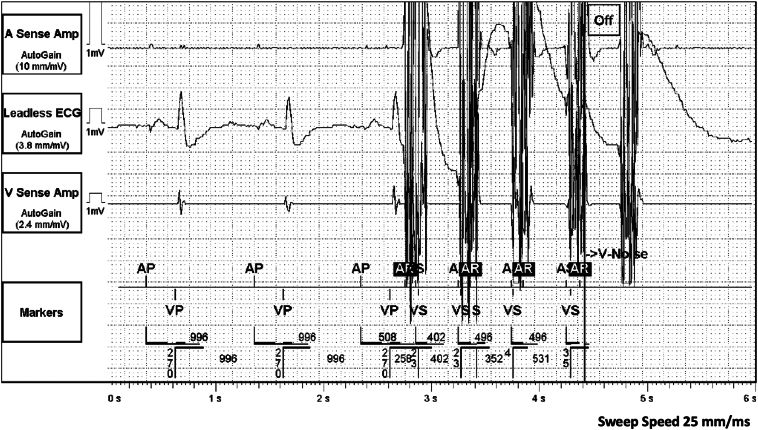
Pulsed electric field application triggering a ventricular noise reversion episode. Shown are high-frequency, large-amplitude pulses characteristic of one pulsed-field ablation application.

During delivery of each lesion, unsynchronized therapeutic waveforms in a hierarchical set of microsecond scale biphasic pulses are delivered.^4^ With the increased use of PFA nationally and internationally, patients with CIEDs are likely to record such artifactual tracings. It is valuable for all clinicians interacting with CIEDs to appreciate these patterns of oversensing when investigating device alerts during interrogations, particularly as these findings are likely to become more prevalent with continued uptake of PFA technologies.

## Disclosures

Joshua Lampert reports a consulting agreement with Viz.ai. Jacob Koruth reports a consulting agreement with Boston Scientific. Vivek Y. Reddy serves as an unpaid consultant to Boston Scientific; and unrelated to this manuscript, he has served as a consultant for and has equity in Ablacon, Acutus Medical, Affera-Medtronic, Anumana, Apama Medical-Boston Scientific, APN Health, Append Medical, Aquaheart, Atacor, Autonomix, Axon Therapies, Backbeat, BioSig, CardiaCare, Cardiofocus, CardioNXT / AFTx, Circa Scientific, CoRISMA, Corvia Medical, Dinova-Hangzhou DiNovA EP Technology, East End Medical, EPD-Philips, EP Frontiers, Epix Therapeutics-Medtronic, EpiEP, Eximo, Farapulse-Boston Scientific, Field Medical, Focused Therapeutics, HRT, Intershunt, Javelin, Kardium, Keystone Heart, Laminar Medical, LuxMed, Medlumics, Middlepeak, Neutrace, Nuvera-Biosense Webster, Oracle Health, Pulse Biosciences, Restore Medical, Sirona Medical, SoundCath, Valcare; unrelated to this work, has served as a consultant for Abbott, Adagio Medical, AtriAN, Biosense-Webster, BioTel Heart, Biotronik, Cairdac, Cardionomic, CoreMap, Fire1, Gore & Associates, Impulse Dynamics, Medtronic, Novartis, Novo Nordisk, Philips; and has equity in Atraverse, DRS Vascular, Manual Surgical Sciences, Newpace, Nyra Medical, Surecor, and Vizaramed. All other authors have no conflicts to disclose.

## References

[1] Swerdlow C.D., Asirvatham S.J., Ellenbogen K.A., Friedman P.A. (2014). Troubleshooting implanted cardioverter defibrillator sensing problems I. Circ Arrhythm Electrophysiol.

[2] Swerdlow C.D., Asirvatham S.J., Ellenbogen K.A., Friedman P.A. (2015). Troubleshooting implantable cardioverter-defibrillator sensing problems II. Circulation Arrhythm Electrophysiol.

[3] Glikson M., Trusty J.M., Grice S.K., Hayes D.L., Hammill S.C., Stanton M.S. (1998). Importance of pacemaker noise reversion as a potential mechanism of pacemaker-ICD interactions. Pacing Clin Electrophysiol.

[4] Reddy V.Y., Anic A., Koruth J. (2020). Pulsed field ablation in patients with persistent atrial fibrillation. J Am Coll Cardiol.

